# Direct effects of adipocyte lipolysis on AMPK through intracellular long-chain acyl-CoA signaling

**DOI:** 10.1038/s41598-023-50903-w

**Published:** 2024-01-02

**Authors:** Abir A. Rahman, Andrew J. Butcko, Emmanuel Songyekutu, James G. Granneman, Emilio P. Mottillo

**Affiliations:** 1 Hypertension and Vascular Research Division, Department of Internal Medicine, Henry Ford Hospital, 6135 Woodward Ave., Detroit, MI 48202 USA; 2 Department of Physiology, Wayne State University School of Medicine, Detroit, MI 48202 USA; 3 Center for Molecular Medicine and Genetics, Wayne State University School of Medicine, Detroit, MI 48202 USA

**Keywords:** Imaging, Biochemistry, Lipids

## Abstract

Long-chain acyl-CoAs (LC-acyl-CoAs) are important intermediary metabolites and are also thought to function as intracellular signaling molecules; however, the direct effects of LC-acyl-CoAs have been difficult to determine in real-time and dissociate from Protein Kinase A (PKA) signaling. Here, we examined the direct role of lipolysis in generating intracellular LC-acyl-CoAs and activating AMPK in white adipocytes by pharmacological activation of ABHD5 (also known as CGI-58), a lipase co-activator. Activation of lipolysis in 3T3-L1 adipocytes independent of PKA with synthetic ABHD5 ligands, resulted in greater activation of AMPK compared to receptor-mediated activation with isoproterenol, a β-adrenergic receptor agonist. Importantly, the effect of pharmacological activation of ABHD5 on AMPK activation was blocked by inhibiting ATGL, the rate-limiting enzyme for triacylglycerol hydrolysis. Utilizing a novel FRET sensor to detect intracellular LC-acyl-CoAs, we demonstrate that stimulation of lipolysis in 3T3-L1 adipocytes increased the production of LC-acyl-CoAs, an effect which was blocked by inhibition of ATGL. Moreover, ATGL inhibition blocked AMPKβ1 S108 phosphorylation, a site required for allosteric regulation. Increasing intracellular LC-acyl-CoAs by removal of BSA in the media and pharmacological inhibition of DGAT1 and 2 resulted in greater activation of AMPK. Finally, inhibiting LC-acyl-CoA generation reduced activation of AMPK; however, did not lower energy charge. Overall, results demonstrate that lipolysis in white adipocytes directly results in allosteric activation of AMPK through the generation of LC-acyl-CoAs.

## Introduction

The hydrolysis of triacylglycerol (TAG) in adipocytes is initiated by the stimulation of adrenergic receptors, leading to activation of Protein Kinase A (PKA) which phosphorylates the resident lipid droplet (LD) protein perilipin 1 (PLIN1)^1^ thereby releasing the lipase co-activator α/β hydrolase domain-containing protein 5 (ABHD5; also known as CGI-58)^2,3^. Once free, ABHD5 binds to and activates Adipose triglyceride lipase (ATGL; also known as Patatin Like Phospholipase Domain Containing 2, PNPLA2), the rate-limiting enzyme for TAG hydrolysis^4,5^. In addition, PKA also phosphorylates hormone-sensitive lipase (HSL) which promotes its translocation to LDs and increases its activity towards diacylglycerol generated by ATGL. Finally, monoacylglycerol (MAG) is hydrolyzed by MAG lipase (MAGL) to a fatty acid and glycerol. The released fatty acids are quickly metabolized to long-chain acyl-CoAs (LC-acyl-CoAs) and can have various fates, such as mitochondrial oxidation or re-esterification into TAG^6,7^.

In addition to fatty acids functioning as metabolic substrates, recent evidence demonstrates that lipolysis can mediate cell signaling events. For example, in adipocytes, the hydrolysis of TAG mobilizes free fatty acids (FFAs) which can function as ligands for peroxisome proliferator-activated receptor (PPAR) nuclear receptors to promote the transcription of genes involved in fatty acid metabolism and mitochondrial function^8,9^. In addition to fatty acids, LC-acyl-CoAs can also regulate metabolism by inhibiting various metabolic enzymes such as lipases^10,11^ and lactate dehydrogenase^12^. In addition, LC-acyl-CoAs are allosteric regulators of ABHD5 that promote its interaction with PLIN1/5, thereby allowing ABHD5 to sense the local pool of LC-acyl-CoAs to limit further fatty acid release^13,14^. These data suggest that lipolysis directly mediates signaling events; however, the direct effects of lipolysis have been difficult to dissociate from receptor-mediated/PKA signaling due to the lack of available tools.

AMP activated kinase (AMPK) is a highly conserved cellular energy sensor in adipocytes that responds to various extracellular and intracellular signals to maintain energy homeostasis^15,16^. AMPK is a heterotrimeric complex that consists of three subunits, a catalytic α-subunit, and regulatory β- and γ-subunits^15^. AMPK maintains energy homeostasis in part through a canonical pathway that senses changes in the intracellular AMP/ATP ratio through the cystathionine-β-synthase (CBS) domains of the γ-subunit. Allosteric activation by AMP binding results in phosphorylation of α-T172 by upstream kinases such as Liver kinase B1 (LKB1) and Calcium/calmodulin-dependent protein Kinase Kinase β (CaMKKβ) and protection from dephosphorylation^17–19^. In addition to sensing energy charge, AMPK also detects nutrients such as glucose, glycogen and fatty acids through a non-canonical pathway^15^. The β-subunit, along with the amino-terminal lobe of the α-kinase domain, make up the allosteric drug and metabolite (ADaM) domain, a ligand binding pocket of AMPK that is thought to bind an endogenous metabolite^20–22^. Interestingly, activation through the ADaM domain can occur independent of α-T172 phosphorylation but requires auto-phosphorylation of S108 on the β1-subunit or phosphorylation by Unc-51-like kinase 1 (ULK1)^23,24^. Overall, the net effect is that AMPK shuts off ATP consuming pathways while increasing ATP producing pathways to maintain energy homeostasis.

AMPK is well known to be activated in adipocytes in response to adrenergic stimulation^25–27^. Previous work suggested that AMPK is activated in adipocytes following consumption of ATP that occurs during lipolysis and subsequent re-esterification; however, the direct effects that lipolysis has on AMPK independent of PKA signaling could not be evaluated^28^. Moreover, re-esterification is known to be reduced during adrenergic stimulation^29^, suggesting that other mechanisms may be involved in activating AMPK in adipocytes.

More recently, LC-acyl-CoAs have been reported to be endogenous ligands of AMPK through the ADaM site^21^, in which AMPK responds to exogenous dietary fatty acids to maintain mitochondrial function^20^. Excess fatty acids can cause lipotoxicity and inflammation^30,31^, thus further understanding the homeostatic mechanisms by which adipocytes limit excess intracellular fatty acids and LC-acyl-CoAs is of importance. Here, we examined the direct effects of lipolysis using direct pharmacological probes that activate lipolysis independent of β-adrenergic/PKA signaling and real-time monitoring of LC-acyl-CoA production to test whether lipolysis could generate intracellular LC-acyl-CoAs to activate AMPK.

## Results

### Direct activation of lipolysis in white adipocytes results in AMPK activation and requires ATGL

**Figure 1:**
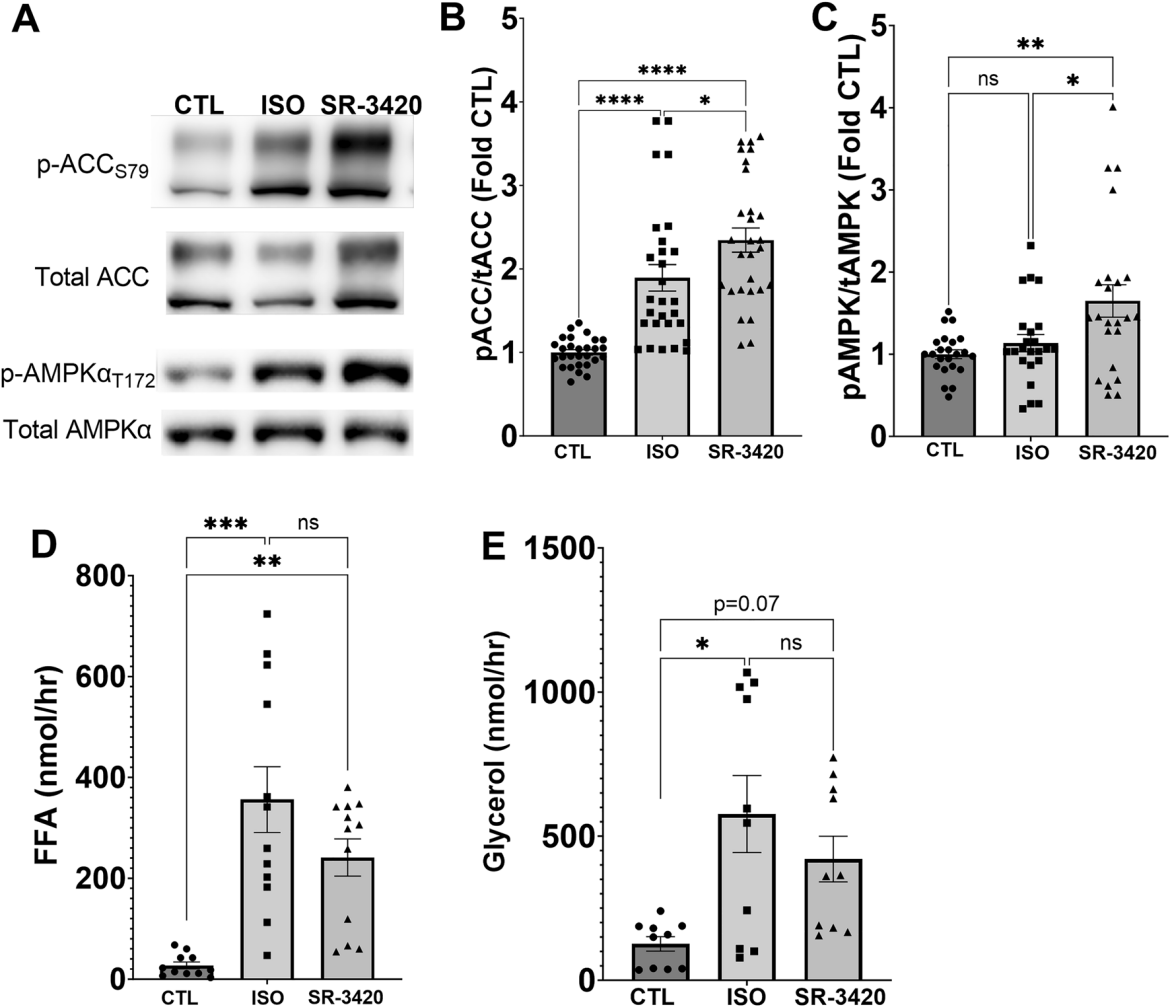
Direct effect of lipolysis on AMPK activation in 3T3-L1 adipocytes. (**A**) Representative western blot images for p-ACC S79, total ACC, p-AMPKα T172 and total AMPKα upon control (CTL) or stimulation of lipolysis by Isoproterenol (ISO) or SR-3420. (**B**) Quantified phospho: total ACC ratio from experiments in (**A**) (n = 10 independent experiments with 2–3 well replicates per experiment). (**C**) Quantified phospho: total AMPK ratio from experiments in A (n = 8 independent experiments with 2–3 well replicates per experiment). FFA (**D**) and glycerol (**E**) in the treatment media following 1 h treatment with Isoproterenol or SR-3420 (n = 4 independent experiments with duplicate wells per experiment). *p > 0.05, **p > 0.01, ***p > 0.001 and ****p > 0.0001 as determined by one-way ANOVA with Tukey’s multiple comparison test.

We initially examined receptor mediated vs direct stimulation of lipolysis on AMPK activation. Stimulation of β-adrenergic receptor with isoproterenol (Iso) resulted in greater phosphorylation of Acetyl CoA Carboxylase (ACC) 1 and 2 on S79, a downstream target of AMPK (Fig. 1A,B). We previously described the development of synthetic ABHD5 ligands which occupy the ligand binding domain of ABHD5 to release it from PLIN1 and activate lipolysis independent of any PKA/receptor signaling in adipocytes^14,32^. Direct stimulation of lipolysis with ABHD5 ligand (SR-3420) also increased ACC phosphorylation and this effect was greater than Iso alone (Fig. 1A,B). While the effect of Iso treatment on AMPKα phosphorylation did not reach statistical significance; SR-3420 significantly increased phosphorylation of AMPKα T172 compared to Basal (CTL) and Iso treatment (Fig. 1B,C). Importantly, both Iso and SR-3420 stimulated lipolysis to similar degrees (Fig. 1D). We wanted to better understand the greater activation of AMPK with direct pharmacological activation of ABHD5 compared to receptor mediated PKA signaling. PKA has been previously shown to phosphorylate and inhibit AMPK on various residue including S485 on AMPKα, which could explain the greater activation of AMPK by SR-3420 in the absence of PKA signaling^33^. Indeed, AMPKα S485 phosphorylation was greatly increased by Iso, but unaffected by SR-3420 (Supplementary Fig. 1A). We confirmed PKA activation by also blotting with a Phospho-PKA substrate antibody, which was increased by Iso, but unaffected by SR-3420 (Supplementary Fig. 1B). Overall, these results demonstrate that direct stimulation of lipolysis independent of receptor mediated/PKA stimulation results in greater activation of AMPK.

**Figure 2:**
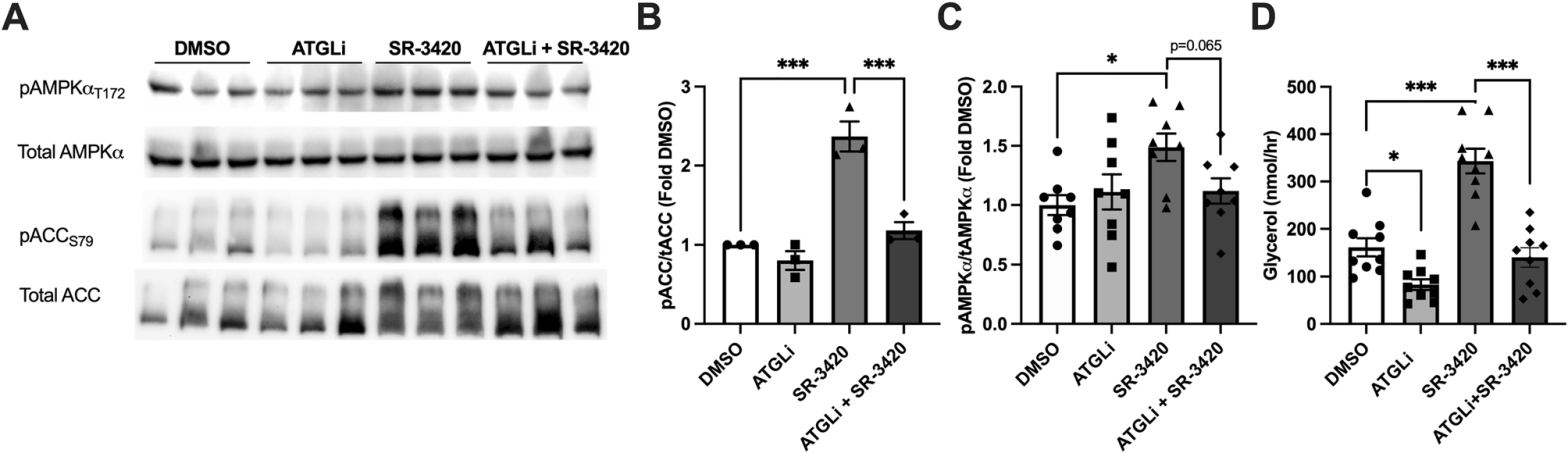
Activation of AMPK through direct stimulation of lipolysis requires ATGL. (**A**) Representative western blot images for p-ACC S79, total ACC, p-AMPKα T172 and total AMPKα following a 30 min pretreatment with either dimethyl sulfoxide (DMSO) control, or Atglistatin (ATGLi; 20 μM) and then a 1 h treatment with lipolysis activator SR-3420 (20 μM) or DMSO. Quantified phospho/total ratios for ACC (**B**) and AMPK (**C**) from experiments in (**A**). (**D**) Glycerol measured in treatment media following 30 min pretreatment with ATGLi and/or 1 h treatment with SR-3420. Data are from n = 3 independent experiments with 2–3 replicate wells per experiment. *p > 0.05, **p > 0.01 and ***p > 0.001 as determined by one-way ANOVA with Šidák’s multiple comparison test.

Next, we tested whether the effects of pharmacological activation of ABHD5 on AMPK required the activity of ATGL, the rate-limiting enzyme for TAG hydrolysis. Direct stimulation of lipolysis with SR-3420 resulted in greater phosphorylation of ACC S79 (Fig. 2A–C) and this effect was reduced by pharmacological inhibition of ATGL (Atglistatin; ATGLi). SR-3420 increased phosphorylation of AMPKα T172 and the effect of ATGL inhibition approached significance (p = 0.065) when compared to the SR-3420 only treated cells. Furthermore, treatment with SR-3420 increased the release of glycerol in the media, a measure of complete TAG hydrolysis, and ATGL inhibition blocked the effect of SR-3420 on glycerol release (Fig. 2D). These results demonstrate that AMPK is activated directly in response to lipolysis through the hydrolase activity of ATGL in adipocytes.

### Activation of lipolysis results in greater production of intracellular LC-acyl-CoAs and phosphorylation of AMPKβ S108

**Figure 3:**
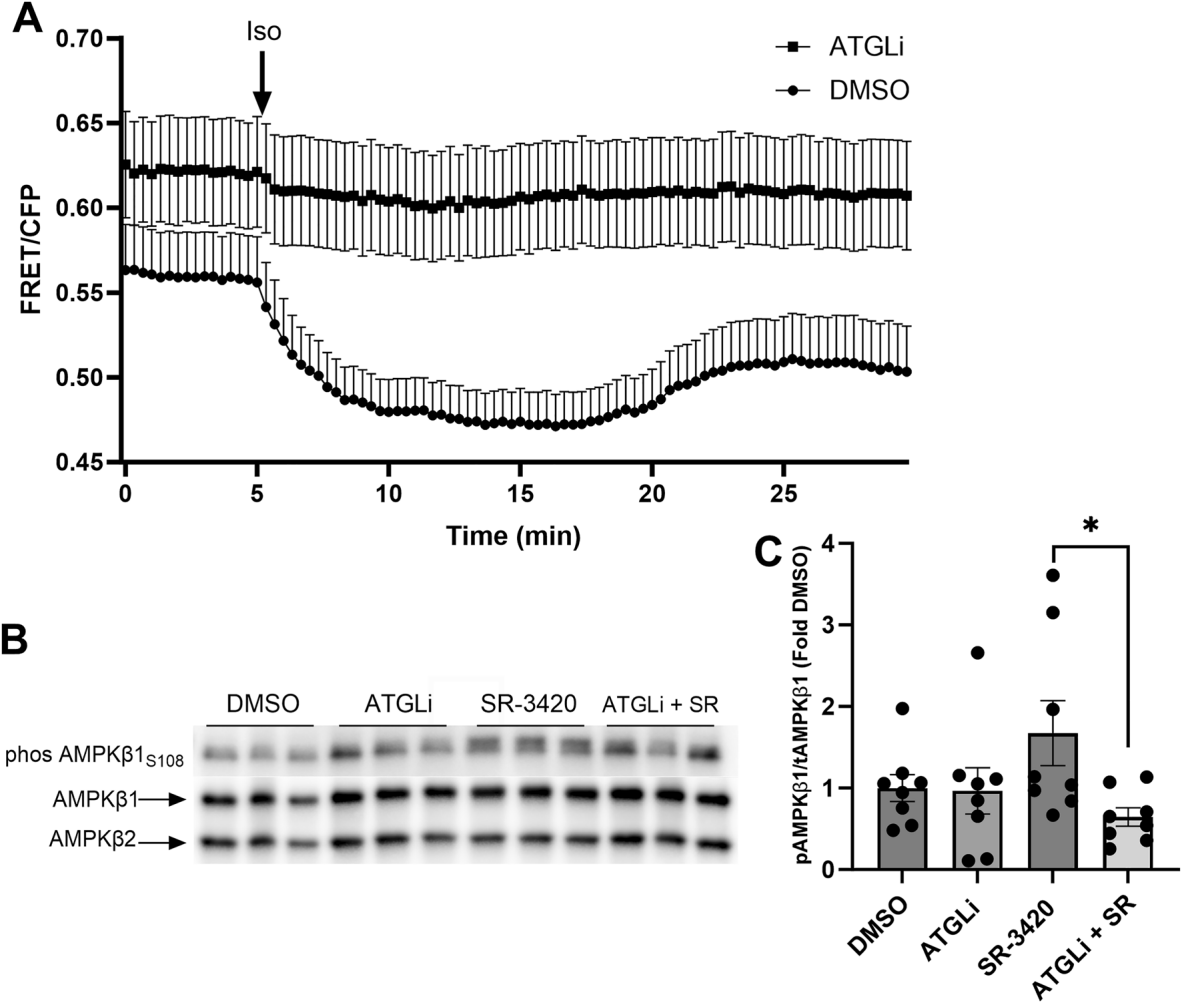
Lipolysis generates LC-acyl-CoAs and promotes AMPK ADaM allosteric activation. (**A**) 3T3-L1 adipocytes stably expressing the LC-acyl-CoA sensor were pretreated with DMSO or Atglistatin (ATGLi, 20 µM) and imaged on a laser scanning confocal every 20 s. Isoproterenol (1 µM) was added at the 5 min mark in between acquisitions to stimulate lipolysis. Data are from one experiment with three coverslips independently imaged for each condition and are representative of 3 individual experiments. (**B**) Representative western blot images for phos-AMPKβ1 S108 and total AMPKβ1/2 in cells pretreated with DMSO or ATGLi, followed by SR-3420 (SR). (**C**) quantified phospho: total AMPKβ1 ratio from experiments in (**B**) (n = 3 independent experiments with 2–3 replicate wells per experiment). *p > 0.05 as determined by one-way ANOVA with Tukey’s multiple comparison test.

LC-acyl-CoAs have recently been proposed to function as endogenous ligands of AMPK through binding of the ADaM site, a function that requires phosphorylation of the β1-subunit of AMPK on S108^20,21^. Moreover, we recently developed a genetically encoded FRET sensor to image intracellular LC-acyl-CoA production in real-time^13^. This reverse FRET sensor relies on the LC-acyl-CoA dependent interaction between ABHD5 and PLIN5^14^ and detects increases in LC-acyl-CoAs by a reduction in FRET^13^. Next, we asked if stimulation of lipolysis in white adipocytes raised intracellular LC-acyl-CoA levels. In 3T3-L1 adipocytes stably expressing the FRET sensor, there was no change in the FRET signal in the initial five minutes (Fig. 3A). Upon stimulation of lipolysis with isoproterenol (Iso), there was a decrease in the FRET ratio, indicating an increase in intracellular LC-acyl-CoAs, an effect that was eliminated with ATGL inhibition (ATGLi; Fig. 3A). Moreover, inhibition of ATGL reduced S108 phosphorylation on the AMPK β1 subunit that was induced by SR-3420 (Fig. 3B,C). These data demonstrate that stimulation of lipolysis results in generation of LC-acyl CoAs and phosphorylation of AMPK β1 S108, effects which are mediated through ATGL.

### Increasing intracellular LC-acyl-CoAs during lipolysis results in greater AMPK activation

**Figure 4:**
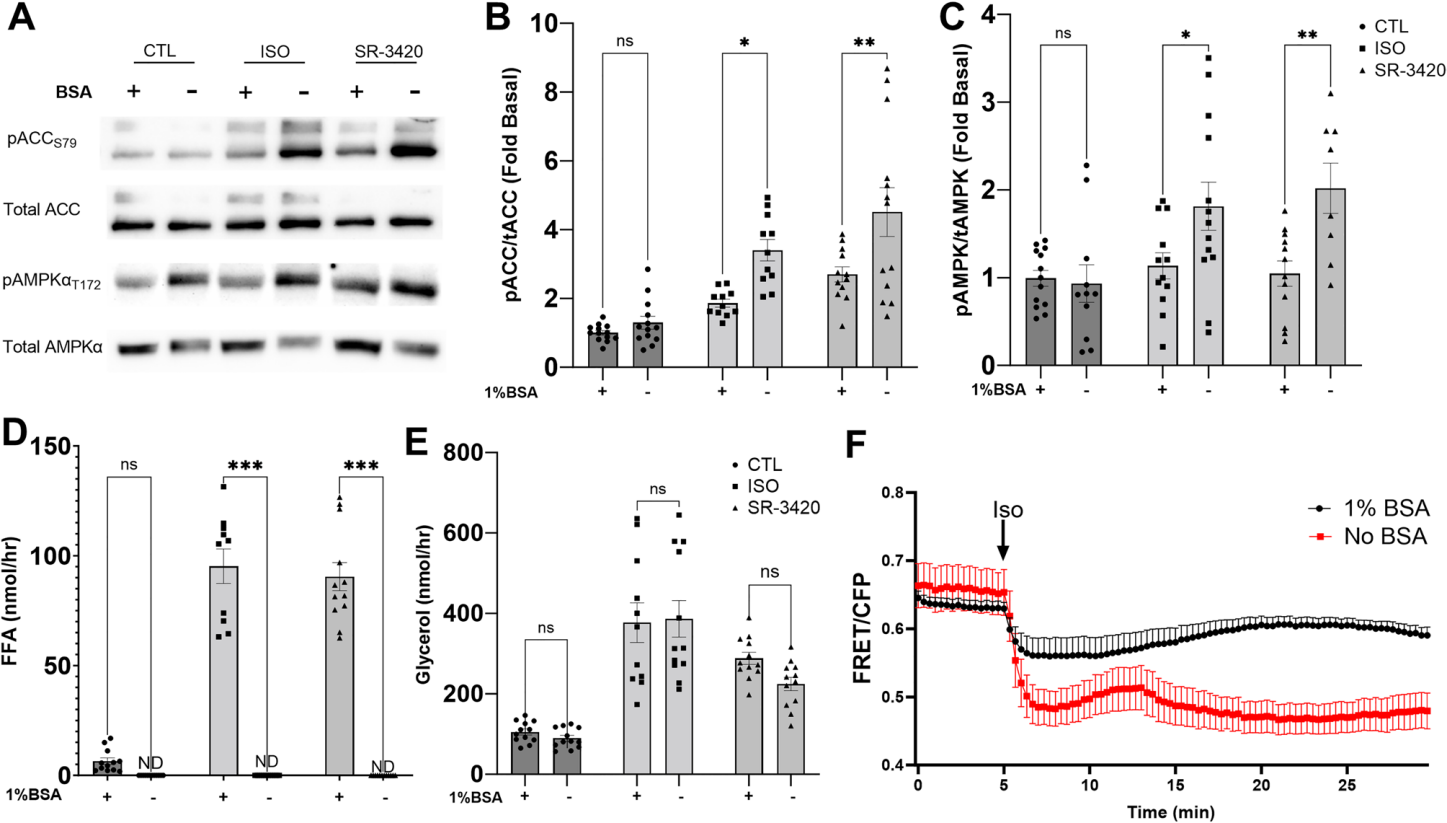
Increasing intracellular LC-acyl-CoAs increases AMPK activation during lipolysis. (**A**) Representative western blot images in 3T3-L1 adipocytes for p-ACC S79, total ACC, p-AMPKα T172 and total AMPKα, treated with Isoproterenol (ISO) or SR-3420, in the presence (+) or absence (−) of 1% BSA in the treatment media. Quantified phospho: total ratio histograms for ACC (**B**) and AMPK (**C**) in 3T3-L1 adipocytes treated in (**A**) (n = 5 independent experiments with 2–3 replicate wells per experiment). FFA (**D**) and glycerol (**E**) in the treatment media following 1 h treatment with Isoproterenol or SR-3420, in the presence ( +) or absence (-) of BSA. (n = 4 independent experiments with duplicates per experiment). *p > 0.05, **p > 0.01, ***p > 0.001 as determined by two-way ANOVA with Tukey’s multiple comparison test. ND; not detectable, ns; not significant. (**F**) FRET imaging of LC-acyl-CoAs in 3T3-L1 adipocytes with or without 1% BSA. 3T3-L1 adipocytes were imaged on a laser scanning confocal every 20 s and Isoproterenol (1 µM) was added at the 5 min mark in between acquisitions to stimulate lipolysis. Data are from one experiment with 2–3 coverslips independently imaged for each condition and are representative of 3 individual experiments.

To further understand if LC-acyl-CoAs are involved in activating AMPK in adipocytes, we manipulated intracellular levels by preventing the efflux of fatty acids with removal of BSA from the media^34^. In response to stimulation of lipolysis by Isoproterenol or SR-3420, the removal of BSA resulted in greater activation of AMPK as indicated by phosphorylation of ACC S79 and AMPK at T172 (Fig. 4A–C). As expected, removal of BSA prevented the efflux of FFA (Fig. 4D) and had no effect on the efflux of glycerol (Fig. 4E). We also examined if the removal of BSA from the media resulted in greater production of LC acyl-CoAs. Stimulation of lipolysis with isoproterenol decreased the FRET ratio as expected, and this effect was greater following removal of BSA from the media (Fig. 4F).

**Figure 5:**
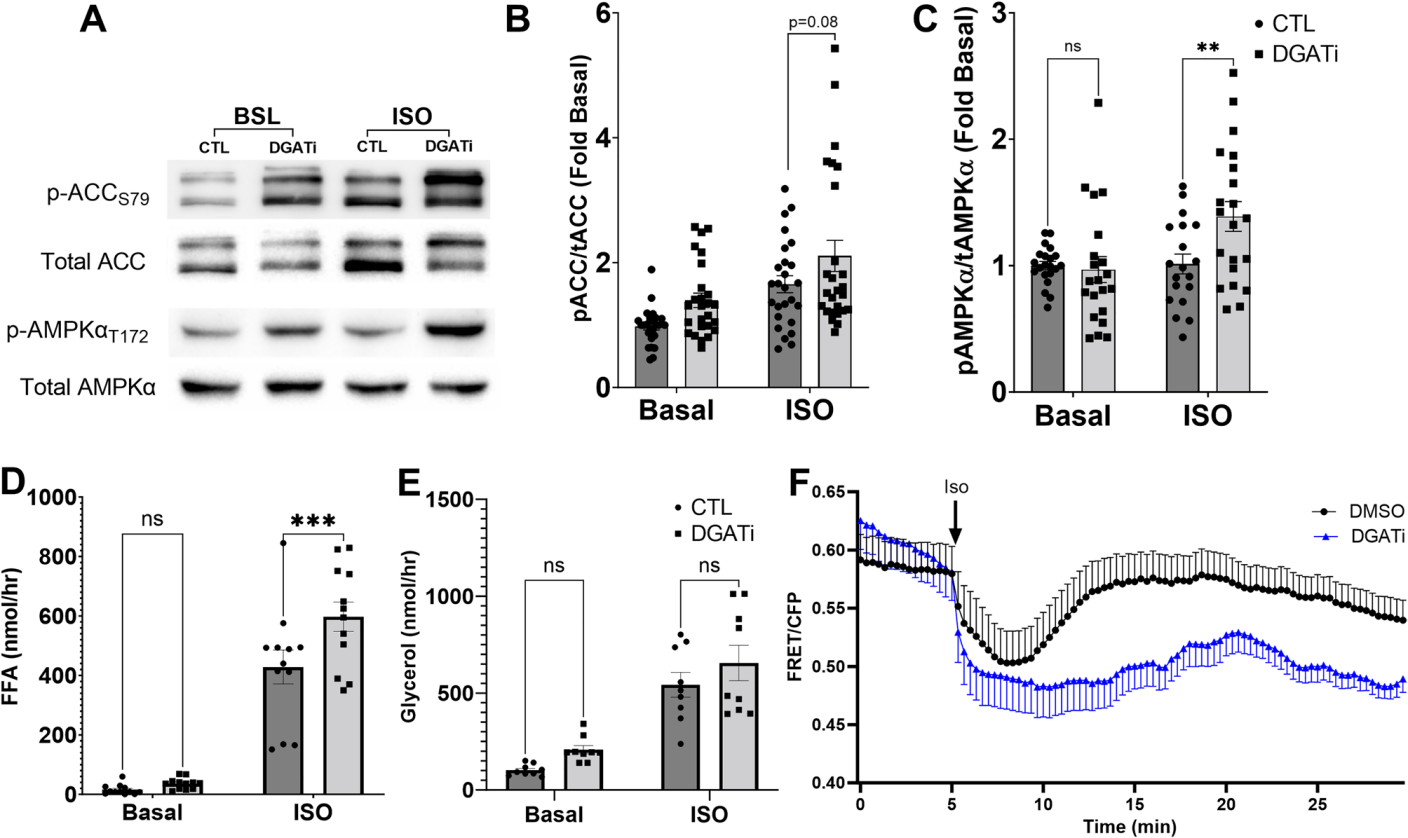
Inhibiting fatty acid re-esterification during lipolysis increases AMPK activity and intracellular LC-acyl-CoAs. (**A**) Representative western blot images for p-ACC S79, total ACC, p-AMPKα T172 and total AMPKα in 3T3-L1 adipocytes pretreated with DGAT inhibitors (DGATi) or DMSO (CTL) followed by control conditions (Basal, BSL) or stimulation with Isoproterenol (ISO) for 1 h. Quantified phospho: total ratio histograms for ACC (**B**) and AMPK (**C**) in 3T3-L1 adipocytes treated in (**A**) (n = 7 independent experiments with 2–3 replicate wells per experiment). FFA (**D**) and glycerol (**E**) in the treatment media as in **A** (n = 3- 4 independent experiments with triplicates per experiment). **p > 0.01, ***p > 0.001 as determined by two-way ANOVA with Tukey’s multiple comparison test, ns; not significant. (**F**) FRET imaging of LC-acyl-CoAs in 3T3-L1 adipocytes pretreated with control (DMSO) or DGAT inhibitors (DGATi), followed by Isoproterenol at 5 min. Data are from one experiment with 3 coverslips independently imaged for each condition and are representative of 3 individual experiments.

As an additional test of the role of LC-acyl-CoA in AMPK signaling we manipulated intracellular LC-acyl-CoA levels by blocking the re-esterification of FFA. As fatty acids are activated by long-chain acyl-CoA synthetase activity, a pool of these LC-acyl-CoAs are re-esterified into TAG by the activity of DGATs^30^. Therefore, we also manipulated LC-acyl-CoA levels by pharmacologically inhibiting the activity of DGAT1 and 2. Treatment of 3T3-L1 adipocytes with DGAT inhibitors (DGAT1 and 2) did not affect basal levels of AMPK phosphorylation. However, under adrenergic stimulation, DGAT inhibition resulted in greater phosphorylation of AMPKα T172 (Fig. 5A,C). The effect of DGAT inhibition on ACC phosphorylation was variable and trended toward statistical significance under isoproterenol stimulation (p = 0.08) (Fig. 5A,B). As expected, DGAT inhibition resulted in greater efflux of fatty acids (Fig. 5D) but did not significantly alter glycerol efflux (Fig. 5E). Importantly, DGAT inhibition elevated intracellular LC acyl-CoA levels when lipolysis was activated by isoproterenol (Fig. 5F). Overall, these data suggest that elevation of intracellular LC-acyl-CoAs augments lipolysis-induced AMPK activation.

### Blocking LC-acyl-CoA generation during lipolysis lowers AMPK activation, but does not prevent changes in energy charge

**Figure 6:**
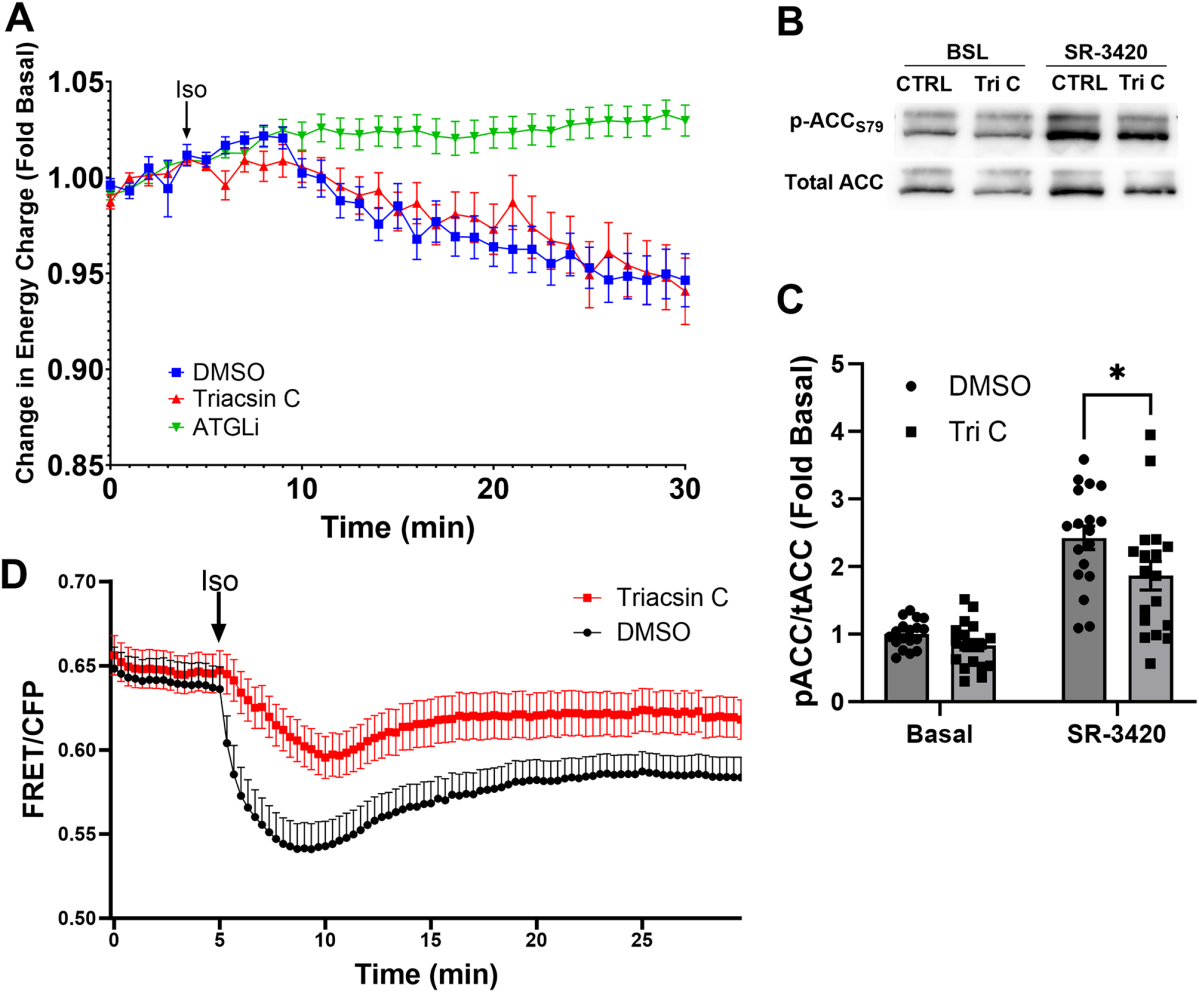
Blocking LC-acyl-CoA generation inhibits AMPK activity. (**A**) 3T3-L1 adipocytes stably expressing the energy charge sensor Perceval were pretreated with control (DMSO), Triacsin C or Atglistatin (ATGLi) for 30 min and imaged on a confocal microscope every minute. Isoproterenol (Iso) was added following pretreatments (DMSO, Triacsin C or ATGLi) and cells were imaged for up to 30 min. Data are from one experiment with 3 coverslips independently imaged for each condition and are representative of 3 individual experiments. (**B**) representative western blot images for p-ACC S79 and total ACC, in 3T3-L1 adipocytes pretreated with Triacsin C (Tri C) followed by direct stimulation of lipolysis with SR-3420 for 1 h. (**C**) Quantified phospho: total ratio histograms for ACC in 3T3-L1 adipocytes treated in (**B**). Data are the averages from 9 independent experiments with duplicate wells per experiment. *p > 0.05; as determined by two-way ANOVA with Šidák’s multiple comparison test. (**D**) FRET imaging of LC-acyl-CoAs in 3T3-L1 adipocytes pretreated with DMSO or Triacsin C for 30 min. 3T3-L1 adipocytes were then imaged on a laser scanning confocal every 20 s and Isoproterenol (1 µM) was added at the 5 min mark in between acquisitions to stimulate lipolysis. Data are from three independent experiment with 2–3 coverslips per experiment.

Recently, Markussun et al. demonstrated that direct stimulation of lipolysis with synthetic ABHD5 ligands reduced ATP levels independent of long-chain acyl-CoA synthetase inhibition^35^, suggesting that the effects of lipolysis on energy charge were independent of fatty acid activation. As such, we investigated the effect of lipolysis on energy charge in live cell imaging of 3T3-L1 adipocytes using the ADP/ATP ratio sensor Perceval^36^. Basal energy charge levels were stable for the first five minutes (Fig. 6A). Isoproterenol reduced the energy charge and this effect was blocked with ATGL inhibition (ATGLi), indicating that this effect is mediated by lipolysis (Fig. 6A). Interestingly, pretreatment with Triacsin C, an inhibitor of LC-acyl-CoA synthetase activity did not prevent the drop in energy charge induced by isoproterenol. Treatment with Oligomycin/Rotenone, inhibitors of ATP synthesis, decreased the energy charge as expected (Supplementary Fig. 2). Direct pharmacological activation of lipolysis with SR-3420 increased ACC S79 phosphorylation and this effect was reduced by Triacsin C (Fig. 6B,C). Importantly, Triacsin C reduced the generation of LC acyl-CoAs by lipolytic stimulation as detected by FRET imaging (Fig. 6D). Overall, these data suggest that the generation of LC-acyl-CoAs results in activation of AMPK, but does not cause a significant change in the energy charge.

## Discussion

Maintenance of lipid homeostasis is critical for cellular function. LC-acyl-CoAs have been recognized as signaling molecules, functioning as allosteric regulators and inhibitors of enzyme activity^12,14^. However, the additional roles that LC-acyl-CoAs play in regulating adipocyte metabolism are poorly defined.

AMPK is an important energy sensor of the cell, that can sense changes in energy charge and nutrients directly though the cystathionine-β-synthase and ADaM domains, respectively^15^. Previous work demonstrated that AMPK is activated in response to changes in energy charge which occurs during lipolysis. Gauthier et al. previously showed that the activation of AMPK was blocked by inhibiting the generation of LC-acyl-CoAs, which utilizes ATP hydrolysis^28^. More recently, LC-acyl-CoAs derived from exogenous dietary fatty acids have been suggested to be endogenous allosteric activators of AMPK in the liver^20,21^. LC-acyl-CoAs are thought to be compartmentalized and channeled to distinct metabolic pathways^37^; however, this has been difficult to prove due to limitations in imaging intracellular LC-acyl-CoAs. We recently developed a FRET sensor to image intracellular LC-acyl-CoAs production in real-time and demonstrated that in brown adipocytes LC-acyl-CoAs produced by mitochondria are sensed by ABHD5 to provide feedback control on lipolysis^13^. In contrast, white adipocytes mostly store and efflux fatty acids with limited ability to oxidize them, suggesting LC-acyl-CoAs may have additional signaling mechanisms. In the current study, we investigated in white adipocytes the direct role of lipolysis in activating AMPK through the production of LC-acyl-CoAs. PKA-independent stimulation of lipolysis with synthetic ABHD5 ligands resulted in the phosphorylation of AMPK β1 S108 subunit, suggesting that lipolysis sensitized AMPK to allosteric activation in the ADaM site. Moreover, manipulating and monitoring intracellular LC-acyl-CoAs in real-time by preventing fatty acid efflux, as well as fatty acid re-esterification, both resulted in greater AMPK activation. While removal of BSA from the media increased intracellular LC-acyl-CoAs levels, this is also known to increase intracellular FFA levels which could alternatively explain the greater activation of AMPK (see discussion below). Thus, a model emerges where upstream signals such as energy charge or upstream kinases (i.e. LKB1 or CaMKK2) could sensitize AMPK during lipolysis to LC-acyl-CoA activation^23^. The direct sensing of LC-acyl-CoAs generated by lipolysis suggests a mechanism by which AMPK senses the nutritional status of the cell to then modify cellular metabolism through the phosphorylation of multiple targets^15^.

The biological significance of TAG cycling as an energy consuming pathway has been contentious and estimates of ATP utilized by lipolysis are a matter of debate as the energetic cost of this cycle is considered to be small^38^. Moreover, the consumption of energy required for de novo fatty acid synthesis that is coupled to lipolysis may represent an alternative pathway by which lipolysis increases ATP demand^39^. In addition, PNPLA enzymes have been recognized to have transacylase activity, allowing them to reacylate TAG independent of CoA, which would allow recycling of fatty acids without ATP consumption^40^. In support, lipase and transacylase activity seem to be intimately linked in ATGL^41^. Recently, advancements in multi-plexing of alkyne fatty acid tracers and mass spec approaches have allowed direct quantification of fatty acid cycling in mouse adipocytes with the re-arrangement of fatty acids having a half-life of 2–4 h and complete exchange of all three FAs on a TAG molecule occurring within 24 h. Here, Wunderling et al. estimated that in 3T3-L1 adipocytes just the storage of TAG would cost 1% of its energy content, an effect that was determined to not be energetically possible^42^, suggesting that there may be ATP independent pathways to TAG remodeling. Moreover, it was suggested that fatty acid exchange allows for continuous editing of TAG molecules in order to alter saturation and remove damaged fatty acids by peroxidation^42^. In addition, re-esterification has been shown to protect adipocytes from fatty acid-induced ER stress^30^.

Interestingly, we observed that direct stimulation of lipolysis with pharmacological activation of ABHD5 resulted in greater activation of AMPK than isoproterenol, although both stimuli resulted in similar rates of lipolysis and re-esterification^32^. Indeed, PKA signaling antagonizes AMPK activity through direct phosphorylation^33^, and we found that Isoproterenol increased PKA signaling and AMPK S485 phosphorylation, a site known to be phosphorylated by PKA. These data further support an inhibitory role for PKA in AMPK signaling during lipolysis.

AMPK subunits have tissue specific expression; however, an understanding of the tissue specificity has been unclear. Interestingly, the AMPK β1 isoform is highly expressed in tissues that store and release fatty acids, such as liver and white and brown adipose tissue and AMPK β1 complexes are sensitive to allosteric activation by LC-acyl-CoAs and ADaM site activation, while β2 is not^21^. Thus, the expression of AMPK β1 in these tissues may represent a mechanism by which AMPK can sense the uptake of fatty acids^20^ and their intracellular accumulation (present study) to maintain lipid homeostasis. Adipocytes express β1 as the major β-subunit isoform^43^ and the phosphorylation of AMPK β1 S108 sensitizes it to allosteric activation of the ADaM site^24^. We found that inhibition of ATGL reduced both the phosphorylation of S108 as well as the production of intracellular LC-acyl-CoAs, suggesting that AMPK senses intracellular LC-acyl-CoAs generated by lipolysis.

Recently, Sharma et al. demonstrated that inhibition of DGAT1 and 2 increased basal AMPK activation in brown adipocytes and that this was associated with an increase in AMP levels that occurred during uncoupling^44^. While we cannot completely rule out changes in energy charge that occur due to direct effects of FFAs on mitochondrial uncoupling^44^ or inhibition of ATP synthase^35,45^, our data support a model in which AMPK senses the cellular environment in adipocytes through multiple mechanisms to maintain energy homeostasis.

## Methods

### Cell culture

3T3-L1 (ATCC CL-173) fibroblast cells were obtained from ATCC and cultured in Dulbecco’s Modified Eagle’s Medium (DMEM), with 4.5 mg/dL glucose and supplemented with 10% Bovine Calf Serum. The cells were differentiated into adipocytes following methods described by Zebisch et al.^46^. All experiments were performed on 3T3-L1 adipocytes on day 11–14 of differentiation with 90% or greater differentiation. For experiments examining AMPK signaling, 3T3-L1 adipocytes grown in six-well plates were placed in 1 ml HEPES Krebs Ringer Buffer (HKRB, 10 mM Hepes pH 7.4) with 1% fatty acid free bovine serum albumin (BSA; Sigma) for 4 h, followed by a change with fresh HKRB 1% BSA containing Control (DMSO), isoproterenol (Iso, 10 µM) or SR-3420 (20 µM). In experiments with pharmacological inhibitors, cells were placed in HKRB 1% BSA for 3.5 h followed by a media change and pretreated with ATGL inhibitor (Atglistatin; 20 µM), long-chain acyl-CoA synthetase inhibitor (Triacsin C; 5 µM), DGAT inhibitors (DGAT1, PF04620110; 1 µM and DGAT2, PF0642439; 5 µM; Cayman Chemical) for 30 min followed by stimulation of lipolysis with isoproterenol (10 µM) or SR-3420 (20 µM) for 1 h.

Lentivirus production and stable cell line generation was performed as previously described^13,47^. Briefly, lentivirus was produced by transfecting HEK293T cells with target plasmid, plus pMD2.G (Addgene # 12259) and psPAX2 (Addgene #12260) packaging vectors using Lipofectamine LTX and Plus reagent (Invitrogen). The virus containing media was collected, filtered and centrifuged at 48,000×*g* for 2 h at 4 °C in a Beckman 25.50 fixed angle rotor. Following centrifugation, the viral pellet was resuspended in OPTIMEM. The 3T3-L1 cells stably expressing the LC-acyl-CoA sensor (pCFP-ABHD5^R299N^-YFP-PLIN5^384–417^), in a doxycycline inducible manner were created by infecting with lentivirus for pINDUCER20^48^ which contained the construct for 24 h followed by G418 selection (1 mg/ml) for 7 days. Stable 3T3-L1 cells expressing the PercevalHR ATP/ADP sensor were infected for 24 h with lentivirus for the PercevalHR ATP/ADP sensor, a gift from Adam Cohen (Addgene plasmid #163061; https://www.addgene.org/163061/; RRID: Addgene_163061).

### Immunoblotting

Following experiments, cells were washed twice in cold DPBS (Sigma) and then lysed in RIPA lysis buffer (Teknova) containing 1% Triton X and 0.1% Sodium dodecyl sulfate, supplemented with phosphatase inhibitors (100 mM NaF, 10 mM Sodium pyrophosphate and 1 mM Na-orthovanadate) and Complete protease inhibitor cocktail (Roche). Samples (1 μg/μL) were prepared in 4 × SDS sample buffer and boiled at 95 °C for 5 min. 20 μg of protein was loaded and Immunoblotting was performed as previously described^16^.

Briefly, western blotting was performed using standard buffers and reagents for SDS-PAGE (Bio-Rad Inc). Proteins were transferred to PVDF membranes (Millipore) and blocked for 1 h in 10 mM Tris (pH 7.6), 137 mM NaCl, 0.1% (vol/vol) Tween 20 (TBST) containing 5% (wt/vol) skimmed milk powder (Research Products International). Membranes were washed with TBST and were incubated in primary antibody dilution buffer (TBST containing 5% wt/vol BSA; Sigma) overnight at 4 °C as using antibodies from Cell Signaling Technology (ACC, #3662; phospho-ACC Ser79/221, #3661; AMPKα, #2532; phospho-AMPKα Thr172, #2535; AMPK β1/β2, #4150; phospho-AMPK β1 S108, #23021; phospho-AMPKα1 S485, #4184; phospho-(Ser/Thr) PKA Substrate, #9621; HRP-linked β-actin, #5125) as recommended by the manufacturer. The antibodies for ACC recognize both ACC2 (280 kDa) and ACC1 (265 kDa) and both bands were quantified. The following day, detection was performed with HRP-conjugated secondary antibodies (anti-Rabbit IgG horseradish peroxidase (HRP)-linked, Cell Signaling Technology #7074) and enhanced chemiluminescence reagent (BioRad). For determination of phospho/total protein levels, immunoblots were first probed for phospho levels, then stripped at 37 °C for 60 min using Restore Plus Western Blot Stripping Buffer (Thermo Fisher Scientific), blocked for 1 h in 5% skimmed milk powder and re-probed overnight to detect total protein levels. In some instances, phospho and total protein levels were determined separately and normalized to loading control (β-actin).

Densitometry was performed using Image J Software (NIH, MD, USA). Each experiment contained 2–3 replicate wells per experiment and the phospho/total ratios were determined. The phospho/total ratios were normalized to an internal control within each experiment (i.e., CTL, DMSO, Basal) to determine the variation between replicates. The normalized averages or individual replicate data from the number of indicated experiments were plotted.

### Live cell confocal imaging

Live cell FRET imaging for LC-acyl-CoAs in 3T3-L1 adipocytes was performed at 37 °C on a laser scanning microscope (Leica SP8) equipped with dual detectors and with continuous Adaptive Focus Control between each frame. 3T3-L1 cells were differentiated in six-well plates containing 25 mm glass coverslips as described above and treated with doxycycline to induce expression of the FRET sensor 24–48 h before imaging. Coverslips were mounted in Attofluor cell chambers (Invitrogen) containing 0.5 ml of 10 mM HEPES Krebs Ringer buffer (Sigma K) and imaged with a 63X 1.40NA Plan-Apochromat objective oil lens (Leica). Cerulean and Citrine were monitored simultaneously with a 405 nm laser excitation and 450–515 nm and 520–600 nm emission, respectively, using dual detectors. Images were captured every 20 s in laser scanning mode with bidirectional scanning. Cells were pretreated with inhibitors as described above. Image analysis was performed using Leica Application Suite X (LAS X) to quantify the regions of interest (i.e., individual cells) for ECFP and EYFP-FRET emission signals and expressed as a FRET ratio (EYFP-FRET/ECFP).

For live cell imaging of energy charge (ATP/ADP) using PercevalHR, cells were grown on cover slips as described above, followed by live cell imaging via fluorescence confocal microscopy with a spinning-disc confocal unit (Olympus IX 81). Images were captured using a 60 × (1.2 NA) plan apo water immersion lens and a Hamamatsu ORCA cooled CCD camera and processed using the Cell Sens Dimensions software (Olympus), followed by analysis and quantification using Image J. PercevalHR was excited sequentially using FITC 485/20 (~ 500 nm) and ECFP 436/20 nm (~ 420 nm) band-pass filters, and both emissions were collected through a 535/30 nm band-pass filter. ATP binding increases the fluorescence for 500 nm excitation, whereas ADP increases the fluorescence for 420 nm excitation^36^. The ROI of individual cells was identified, and the emission signal intensities were quantified for the excitations at the 500 and 420 nm and the ratio was calculated (500/420) as a measure of the intracellular ATP:ADP ratio.

### Measurement of fatty acid and glycerol release

Free fatty acids and glycerol released in the media were measured on a BioTek Synergy H1 microplate reader as previously detailed^49^. Free fatty acid release was determined using a NEFA-HR kit (Fujifilm) adapted to fluorescence detection with Amplex Red (10-Acetyl-3,7-dihydroxyphenoxazine, Cayman Chemical) and the fluorescence was read for Resorufin with excitation at 554 nm and emission at 593 nm. Glycerol release was determined using Free Glycerol Reagent (Sigma) and the absorbance was measured at 540 nm.

### Statistical analysis

All data are reported as mean ± SEM. Statistical significance was determine using GraphPad Prism software version 10. Data were analyzed with One-way ANOVA followed by Tukey’s or Šidák’s multiple comparison test or with Two-Way ANOVA with Šidák’s multiple comparison test as indicated.

### Supplementary Information

Supplementary Information.

## Data Availability

All data reported in this paper will be shared by the corresponding contact (emottil1@hfhs.org). SR-3420 is a derivative of thiaza-tricyclic urea (TTU). The structure of SR-3420 has previously published^32^ and SR-4995 is a similarly structured TTU and ABHD5 ligand and can be commercially purchased from Sigma Aldrich (Cat# SML2207).

## Abbreviations

ABHD5: Alpha–beta hydrolase domain-containing 5
ATGLi: Atglistatin
ATGL: Adipose triglyceride lipase
BSA: Bovine serum albumin
CaMKKβ: Calcium/calmodulin-dependent protein Kinase Kinase β
DAG: Diacylglycerol
Dox: Doxycycline
FA: Fatty acid
FFA: Free fatty acid
FRET: Fluorescent resonance energy transfer
HSL: Hormone-sensitive lipase
LD: Lipid droplet
LKB1: Liver kinase B1
LC-acyl-CoAs: Long-chain acyl-CoAs
MAG: Monoacylglycerol
PKA: Protein kinase A
PLIN1: Perilipin-1
PNPLA2: Patatin Like Phospholipase Domain Containing 2
PPARs: Peroxisome proliferator-activated receptors
ULK1: Unc-51-like kinase 1
TAG: Triacylglycerol
