# Supplementary material for: Direct effects of adipocyte lipolysis on AMPK through intracellular long-chain acyl-CoA signaling
Source: Sci Rep. 2024 Jan 2;14:19. doi: 10.1038/s41598-023-50903-w (PMC10761689; doi:10.1038/s41598-023-50903-w)
Supplement: Supplementary file 1 — Supplementary Information. [file 41598_2023_50903_MOESM1_ESM.pdf]

Direct effects of adipocyte lipolysis on AMPK through intracellular Long-chain acyl-CoA signaling

Abir A. Rahman, Andrew J. Butcko, Emmanuel Songyekutu, James G. Granneman and Emilio P. Mottillo

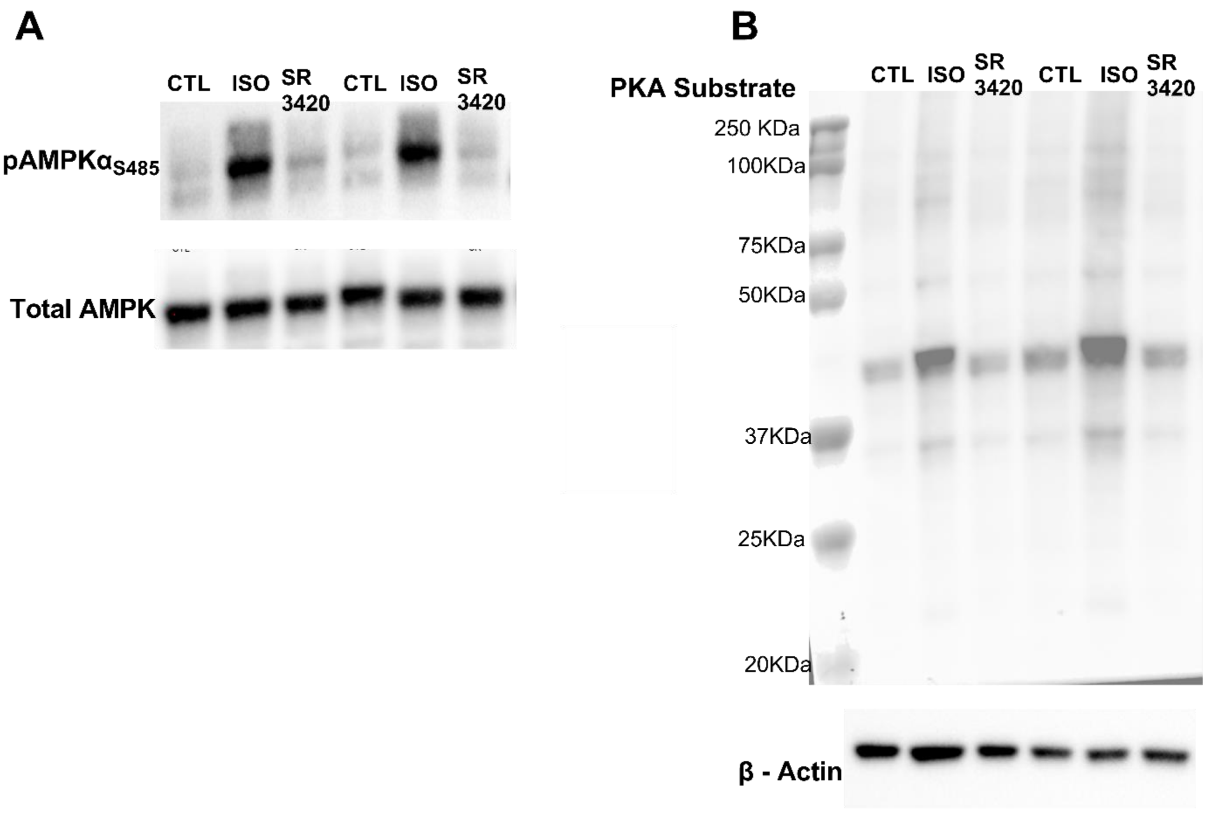

Supplementary Figure 1. Isoproterenol increases AMPK S485 phosphorylation and PKA signaling but SR-3420 does not. **A**, Replicate sample wells from Figure 1 were blotted with pAMPKα1 S485 primary antibody and detected as shown. The blot was then stripped and reblotted with Total AMPK primary antibody. **B**, Replicate samples from the same experiment were blotted with pPKA substrate antibody and then detected. The blot was then stripped and reblotted with β-actin. Data are from one experiment are a representative of four independent experiments.

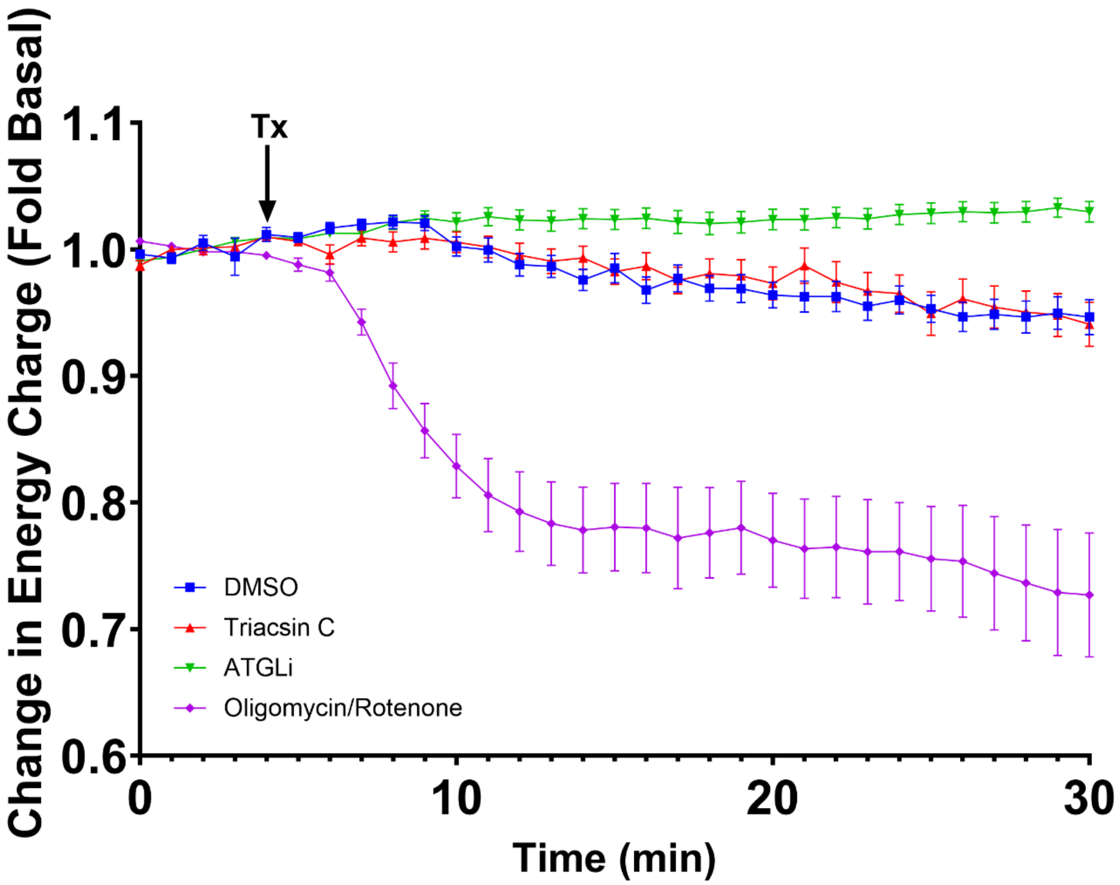

Supplementary Figure 2. Energy charge imaging in 3T3-L1 adipocytes stably expressing the energy charge sensor Perceval. Data corresponds to Figure 6A with the additional inclusion of data for oligomycin (1 µM)/rotenone (2.5 µM) to block ATP synthase and mitochondrial complex I in order reduce energy charge. Iso or oligomycin/rotenone were added at the indicated treatment time (Tx).

Figure 1A Uncropped Blots

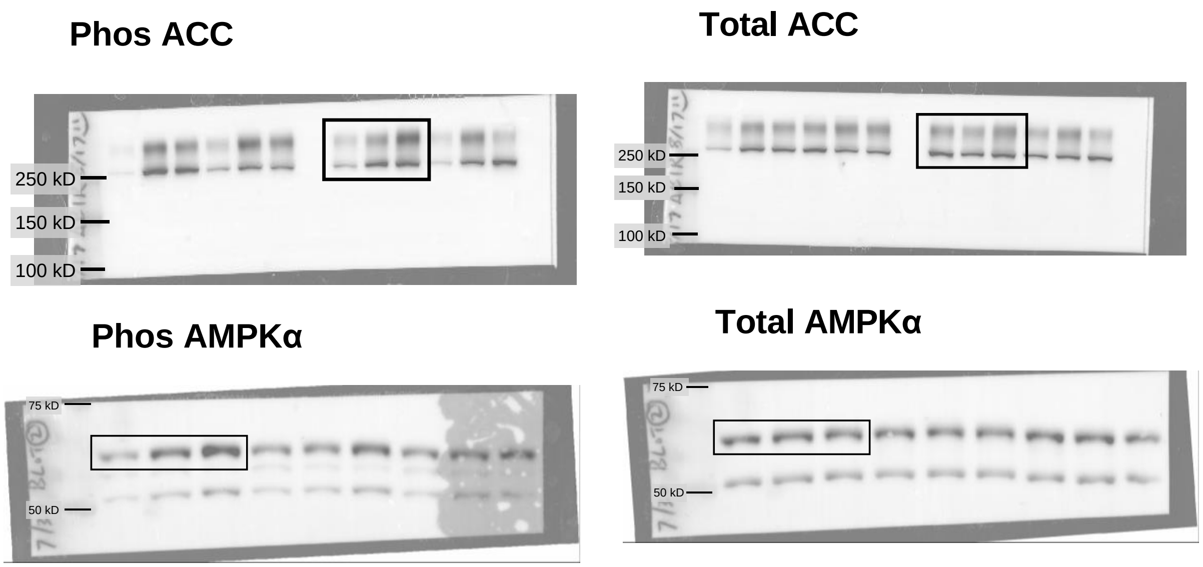

Uncropped images of western blot panels in main figure 1. Black boxes indicate the cropped portion of each immunoblot presented in the corresponding main figure. For the ACC blots, the membranes were cut just below 100 kD before hybridization with primary antibodies. For the AMPKα blots, membranes were cut between 75 kD and 37 kD before hybridization with primary antibodies.

Figure 2A uncropped blots

Phos AMPKα

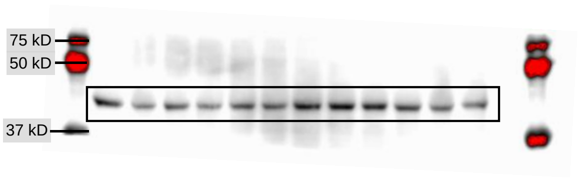

Total AMPKα

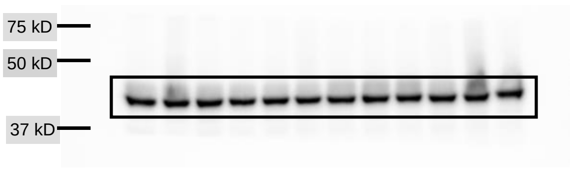

Phos ACC

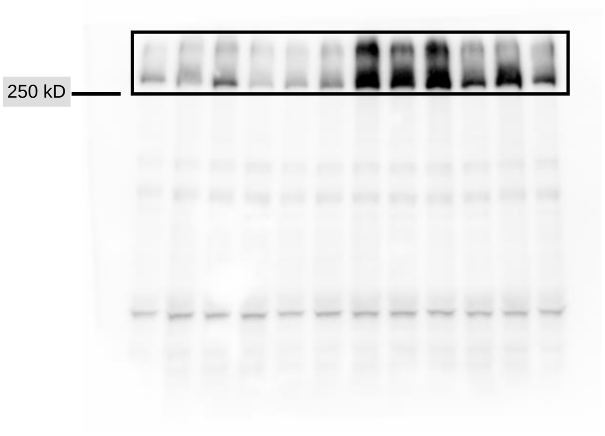

Total ACC

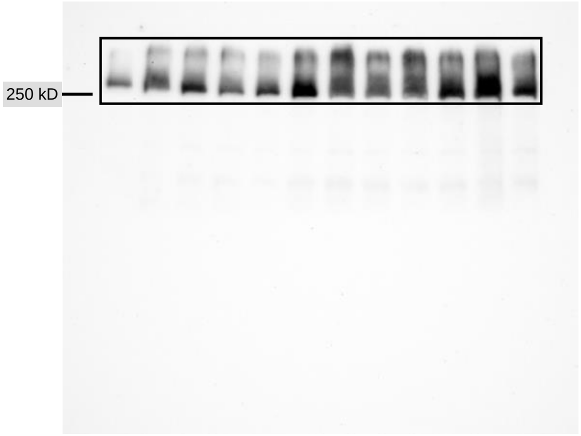

Uncropped images of western blot panels in main figure 2. Black boxes indicate the cropped portion of each immunoblot presented in the corresponding main figure. For the AMPKα blots, membranes were cut between 75 kD and 37 kD before hybridization with primary antibodies.

Figure 3B uncropped blots

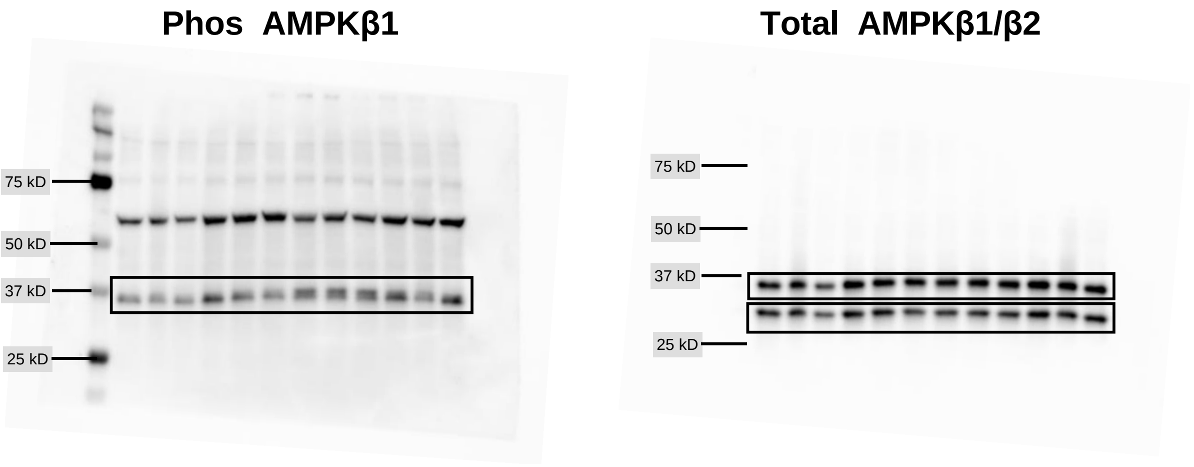

Uncropped images of western blot panels in main figure 3. Black boxes indicate the cropped portion of each immunoblot presented in the corresponding main figure.

Figure 4A uncropped blots

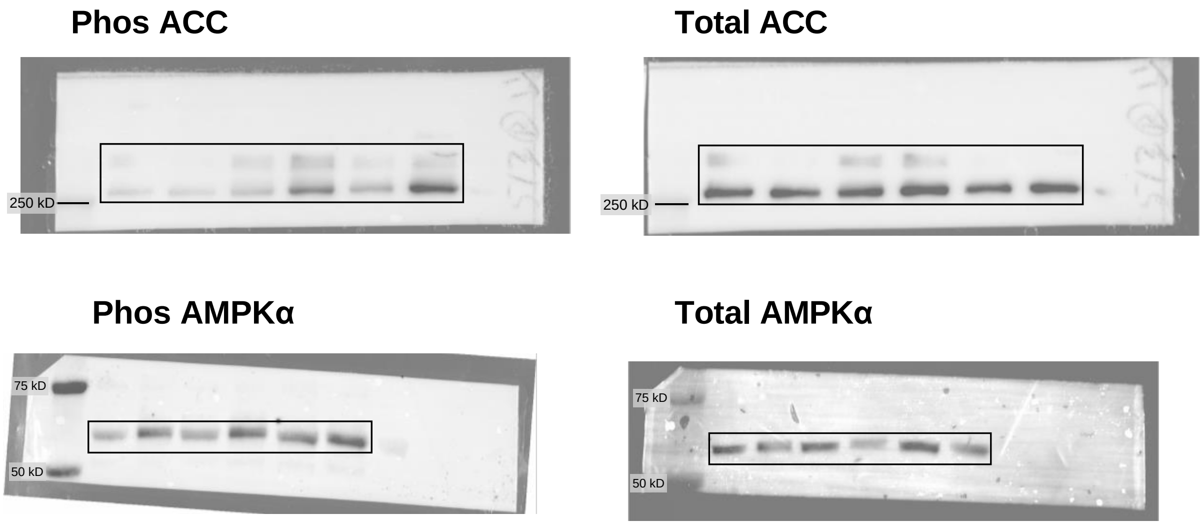

Uncropped images of western blot panels in main figure 4. Black boxes indicate the cropped portion of each immunoblot presented in the corresponding main figure. ACC probed membranes were cut just below 250 kD, while AMPK probed membranes were cut between 75 kD and 50 kD before hybridization with primary antibodies.

Figure 5A uncropped blots

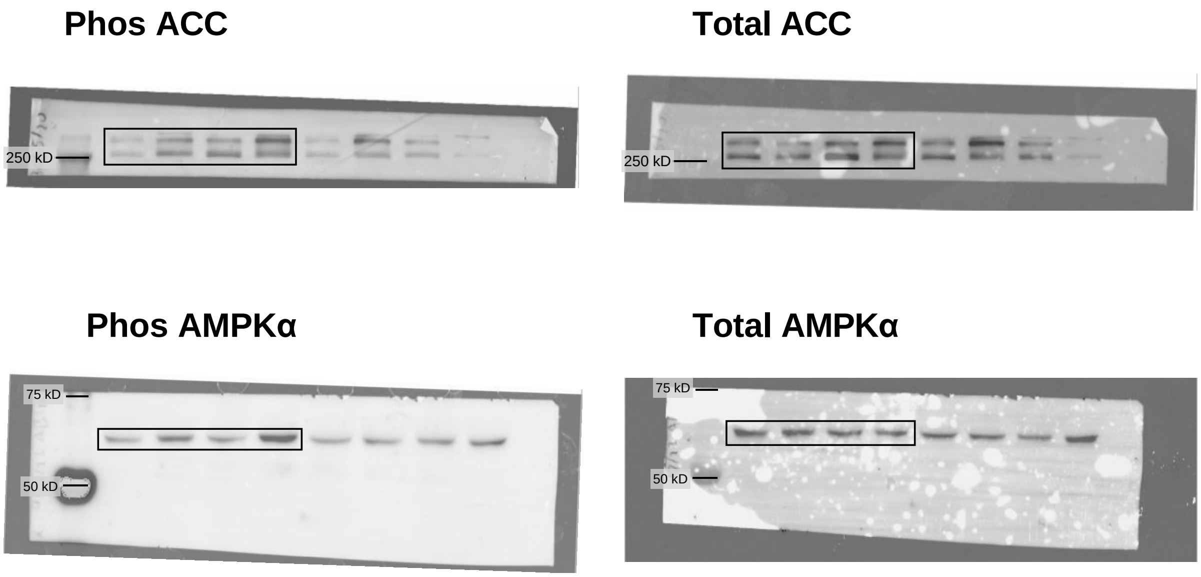

Uncropped images of western blot panels in main figure 5 Black boxes indicate the cropped portion of each immunoblot presented in the corresponding main figure. ACC probed membranes were cut just below 250 kD, while AMPK probed membranes were cut between 75 kD and 50 kD before hybridization with primary antibodies.

Figure 6B uncropped blots

Phos ACC

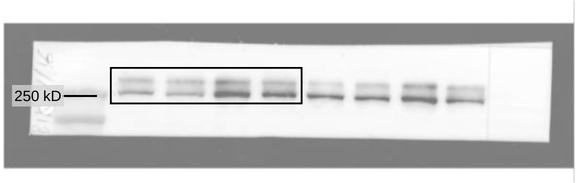

Total ACC

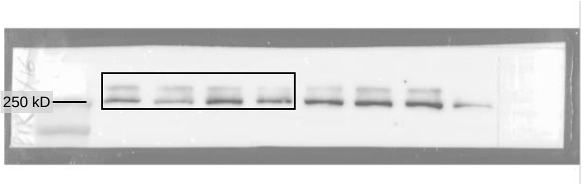

Uncropped images of western blot panels in main figure 6. Black boxes indicate the cropped portion of each immunoblot presented in the corresponding main figure. Membranes were cut just below 250 kD before hybridization with primary antibodies.

Supplemental Figure 1 uncropped blots

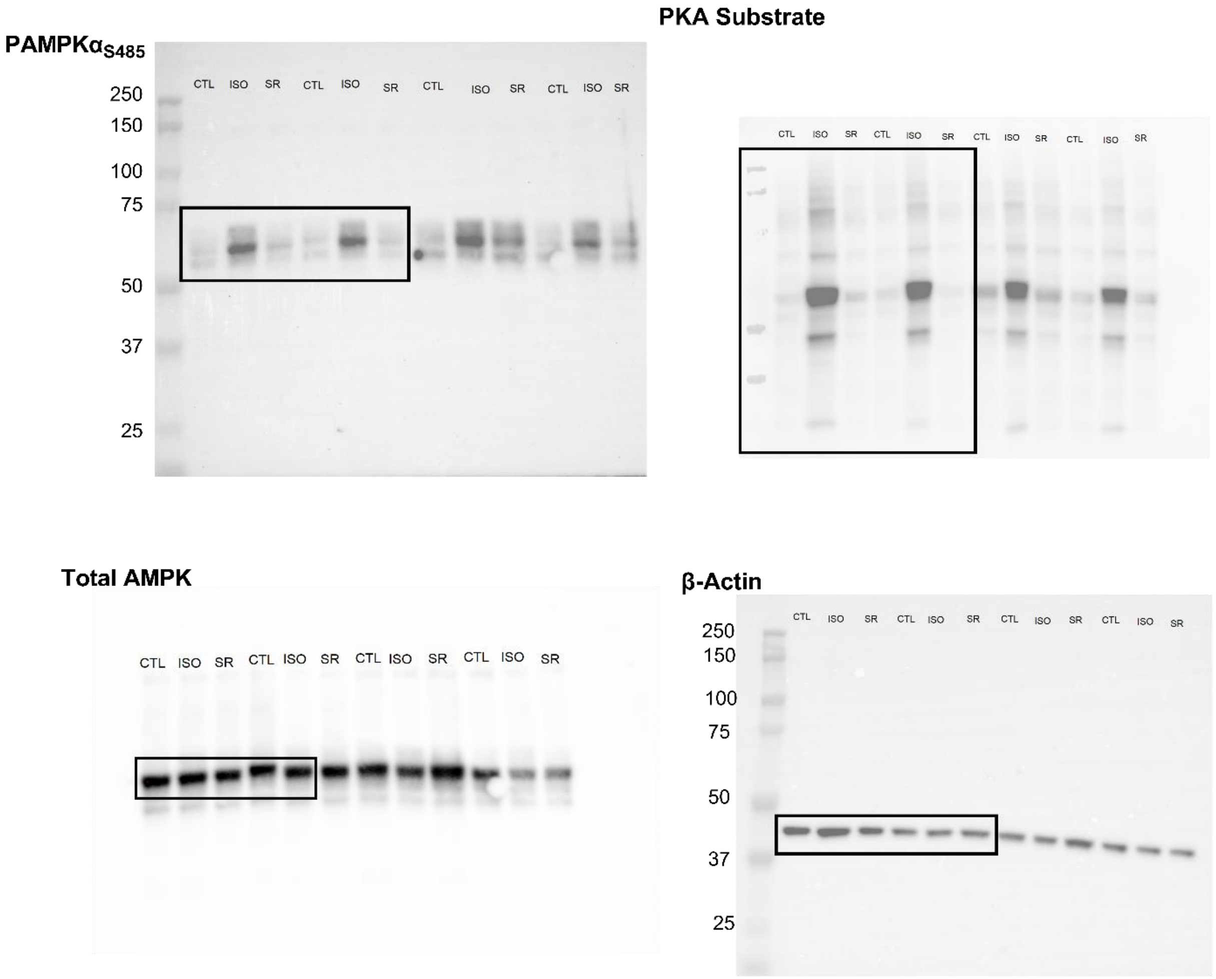

Uncropped images of western blot panels in supplemental figure 1. Black boxes indicate the cropped portion of each immunoblot presented in the corresponding main figure.
